# Optimal control of the COVID-19 pandemic: controlled sanitary deconfinement in Portugal

**DOI:** 10.1038/s41598-021-83075-6

**Published:** 2021-02-10

**Authors:** Cristiana J. Silva, Carla Cruz, Delfim F. M. Torres, Alberto P. Muñuzuri, Alejandro Carballosa, Iván Area, Juan J. Nieto, Rui Fonseca-Pinto, Rui Passadouro, Estevão Soares dos Santos, Wilson Abreu, Jorge Mira

**Affiliations:** 1 Department of Mathematics, Center for Research and Development in Mathematics and Applications (CIDMA), University of Aveiro, 3810-193 Aveiro, Portugal; 2 Department of Physics, Institute CRETUS, Group of Nonlinear Physics, Universidade de Santiago de Compostela, 15782 Santiago de Compostela, Spain; 3 Departamento de Matemática Aplicada II, E. E. Aeronáutica e do Espazo, Campus de Ourense, Universidade de Vigo, 32004 Ourense, Spain; 4 Instituto de Matemáticas, Universidade de Santiago de Compostela, 15782 Santiago de Compostela, Spain; 5 Center for Innovative Care and Health Technology (ciTechCare), Polytechnic of Leiria, Leiria, Portugal; 6 ACES Pinhal Litoral-ARS Centro, Leiria, Portugal; 7 School of Nursing and Research Centre “Centre for Health Technology and Services Research/ESEP-CINTESIS”, Porto, Portugal; 8 Departamento de Física Aplicada, Universidade de Santiago de Compostela, 15782 Santiago de Compostela, Spain

**Keywords:** Control theory, Differential equations, Dynamic networks, Dynamical systems, Population dynamics, Infectious diseases, Applied mathematics

## Abstract

The COVID-19 pandemic has forced policy makers to decree urgent confinements to stop a rapid and massive contagion. However, after that stage, societies are being forced to find an equilibrium between the need to reduce contagion rates and the need to reopen their economies. The experience hitherto lived has provided data on the evolution of the pandemic, in particular the population dynamics as a result of the public health measures enacted. This allows the formulation of forecasting mathematical models to anticipate the consequences of political decisions. Here we propose a model to do so and apply it to the case of Portugal. With a mathematical deterministic model, described by a system of ordinary differential equations, we fit the real evolution of COVID-19 in this country. After identification of the population readiness to follow social restrictions, by analyzing the social media, we incorporate this effect in a version of the model that allow us to check different scenarios. This is realized by considering a Monte Carlo discrete version of the previous model coupled via a complex network. Then, we apply optimal control theory to maximize the number of people returning to “normal life” and minimizing the number of active infected individuals with minimal economical costs while warranting a low level of hospitalizations. This work allows testing various scenarios of pandemic management (closure of sectors of the economy, partial/total compliance with protection measures by citizens, number of beds in intensive care units, etc.), ensuring the responsiveness of the health system, thus being a public health decision support tool.

## Introduction

COVID19 is an ongoing global concern. On March 11, 2020, the World Health Organization (WHO) declared the state of pandemic due to SARS-COV2 infection and, worldwide, the containment strategies to control the spread of COVID-19 were gradually intensified. In the first three months after COVID-19 emerged, nearly 1 million people were infected and 50,000 died. Although we had in the past similar diseases caused by the same family of virus (e.g., SARS and MERS), these strategies are still of huge importance as the rate of spread of the SARS-COV2 virus is higher^1^. The social and clinical experience with COVID-19 will leave lasting marks in society and in the health system, from Latin cultural habits (proximity, touch, kiss) until health system configuration changes, leaving hospitals for more complex clinical situations and providing community institutions (Health Centers, Family Health Units and Integrated Continuous Care Units) with diagnostic and therapeutic means that avoid systematic recourse to hospital emergencies.

By August 15, 2020, the cumulated number of confirmed cases by COVID-19 was of 21,387,974, with 14,169,695 recovered cases and 764,112 deaths, corresponding to 6,454,140 active cases (at a given time *t*, the term “active infected” corresponds to the number of confirmed infected individuals active at that time *t*, while the term “confirmed infected” corresponds to the accumulated number of confirmed infected individuals from the beginning of the epidemic till time *t*). Regarding the active cases, 6,035,791 (99%) suffer mild condition of the disease and 65,488 (1%) are in serious or critical health situation^2^. In Portugal, the first confirmed 2 infected cases were reported on March 2, 2020, and the Government ordered public services to draw up a contingency plan in line with the guidelines set by the Portuguese Public Health Authorities. On March 12, 2020, it was declared State of Emergency. In the following week, additional measures were adopted, such as: prohibition of events, meetings or gathering of people, regardless of reason or nature, with 100 or more people; prohibition of drinking alcoholic beverages in public open-air spaces, except for outdoor areas catering and beverage establishments, duly licensed for the purpose; documentary control of people in borders; the suspension of all and any activity of stomatology and dentistry, with the exception of proven urgent situations and non-postponable. Teaching as well as non-teaching and classroom training activities were suspended from 16th March 2020^3^; the air traffic to and from Portugal was banned for all flights to and from countries that do not belong to the European Union, with certain exceptions. Actually, the Portuguese were advised to stay at home, avoiding social contacts, since 14th March 2020, inclusive, restricting to the maximum their exits from home. From March 20 on, it was mandatory to adopt the teleworking regime, regardless of the employment relationship, whenever the functions in question allow. On May 2 the emergency status was canceled (duration of 45 days). After the 45 days of state of emergency, the Government progressively established measures for the reopening of the economy but with rules for the control of the spread of the virus. Portugal is still in situation of alert, and the situation of calamity and contingency can be declared, depending on the region and the number of active cases. According to the Portuguese Health Authorities, as of the writing, there has not been an overload of intensive care services; since the beginning of the Portuguese outbreak the intensive medicine capacity increased from 629 to 819 beds (+23%) (data from June 14, 2020); the health authorities objective is to reach, by the end of 2020, a ratio of 9.4 beds per 100 thousand inhabitants. Moreover, Portugal did not enter a rupture situation; at the peak of the epidemic (in the end of April, beginning of May), there were 1026 intensive care beds; the levels of intensive medicine occupancy, by June 14, 2020, were of 61% at national level and 65% in the Lisbon and Vale do Tejo region^4^.

The way we manage today the pandemic is related to the ability to produce quality data, which in turn will allow us to use the same data for mathematical modeling tasks, that are the best framework to deal with upcoming scenarios^5^. Many efforts have been done in this field^6–9^. The adjustment of the model parameters in a dynamic way, through the imposition of limits on the system in order to optimize a given function, can be implemented through the theory of optimal control^10^.

The usefulness of optimal control in epidemiology is well-known: while mathematical modeling of infectious diseases has shown that combinations of isolation, quarantine, vaccination and/or treatment are often necessary in order to eliminate an infectious disease, optimal control theory tell us how they should be administered, by providing the right times for intervention and the right amounts^11,12^. This optimization strategy has also been used in some works within the scope of COVID-19. Optimal control of an adapted Susceptible–Exposure–Infection–Recovery (SEIR) model has been done with the aim to investigate the efficacy of two potential lockdown release strategies on the UK population^13^. Other COVID-19 case studies include the use of optimal control in USA^14^. Optimal administration of an hypothetical vaccine for COVID-19 has been also investigated^15^; and an expression for the basic reproduction number in terms of the control variables obtained^16^. According to the most recent pandemic spreading data, until a large immunization rate is achieved (ideally by a vaccine), the application of so-called nonpharmaceutical interventions (NPIs) is the key to control the number of active infected individuals^17^.

Here we are interested in using optimal control theory has a tool to understand ways to curtail the spread of COVID-19 in Portugal by devising optimal disease intervention strategies. Moreover, we take into account several important issues that have not yet been fully considered in the literature. Our model allows the application of the theory of optimal control, to test containment scenarios in which the response capacity of health services is maintained. Because the pandemic has shown that the public health concern is not only a medical problem, but also affects society as a whole^18^, the dynamics of monitoring the containment measures, that allow each individual to remain in the protected *P* class, is here obtained through models of analysis of social networks, which differentiates this study getting closer to the real behavior of individuals and also predicting the adherence of the population to possible government policies.

## Results

### Confirmed active infected individuals in Portugal

We propose a deterministic *SAIRP* mathematical model for the transmission dynamics of SARS-CoV-2 in a homogeneous population, which is subdivided into five compartments depending on the state of infection and disease of the individuals (see Supplementary Fig. 1): *S*, susceptible (uninfected and not immune); *A*, infected but asymptomatic (undetected); *I*, active infected (symptomatic and detected/confirmed); *R*, removed (recovered and deaths by COVID-19); *P*, protected/prevented (not infected, not immune, but that are under protective measures).

The class *P* represents all individuals that practice, with daily efficacy, the so-called non-pharmaceutical interventions (NPIs), e.g., physical distancing, use of face masks, and eye protection to prevent person-to-person transmission of SARS-CoV-2 and COVID-19. Based on recent literature^19,20^, we assume that the individuals in the class *P* are free from infection, but are not immune and, if they stop taking these measures, they become susceptible again, at a rate $\omega = w m$, where *w* represents the transition rate from protected *P* to susceptible *S* and *m* represents the fraction of protected individuals that is transferred from *P* to *S* class (see Supplementary Fig. 1 for the diagram of the model; for the equations and a description of the parameters, see the “Methods” section).

**Figure 1:**
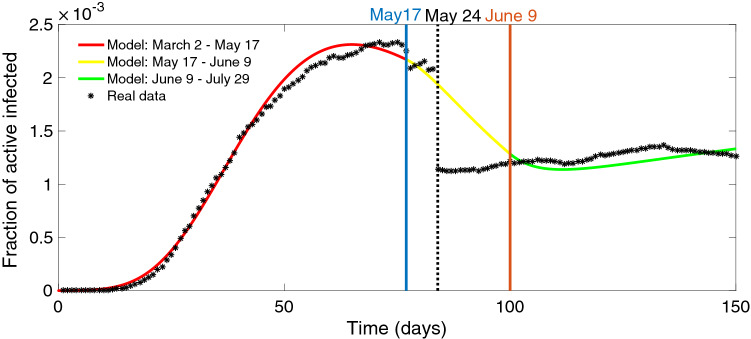
Fraction of confirmed active cases per day in Portugal. Red line: from March 2 to May 17, 2020. Yellow line: from May 17 to June 9, 2020. Green line: from June 9 to July 29, 2020. The drastic jump down in the real data (black points) corresponds to the day when the Portuguese authorities announced 9844 recovered individuals on May 24.

In Fig. 1, we show that the SAIRP model (as described above and in detail in “Methods”) fits well the confirmed active infected cases in Portugal from March 2, 2020 until July 29, 2020 (a total of 150 days), using the data from The Portuguese Public Health Authorities^21^. More precisely, based on daily reports from the Portuguese Public Health Authorities, that provide information about the confirmed infected cases, recovered, and deaths, the active cases are therefore the result of subtracting to the cumulative confirmed cases the sum of the recovered and deaths by COVID-19. See section “Methods” for the parameter values and initial conditions used, as well as their justification.

Most of the parameter values of the *SAIRP* model are fixed for the 150 days considered. However, we analyzed the model in three different time intervals from the first confirmed case, on March 2, until July 29, and the parameters $\beta$, *p* and *m* take different values in these three time intervals. At first, we consider the time interval going from the first confirmed infected individual (March 2) until May 17, that is, 15 days after the end of the three Emergency States in Portugal. Here, despite the fraction of susceptible individuals *S* that are transferred to class *P* being $p_1=0.675$ (see Table 3 in “Methods”), meaning that approximately $67,5\%$ of the population was *protected* due to the COVID-19 confinement policies during the three emergency states (suspension of activities in schools and universities, high risk groups protection and teleworking regime adoption)^21,22^, the number of infected individuals increased exponentially (red curve in Fig. 1). The second time interval goes from May 17 until June 9, the period when the number of new infected individuals grows slower comparing with the beginning of the outbreak. In this time period, and after the end of the three emergency states (during 45 days), the fraction of susceptible individuals that could stay *protected* decreased ($p_2=0.55$), which, together with a low rate of $\beta _2=0.55$, explains the progressive decrease of *I* (yellow curve in Fig. 1). Finally, the model was applied to the period going from June 9 until July 29, 2020. In that case, with the gradual opening of the society and economy, the value for $p_3$ becomes smaller and $\beta _3$ increases as the number of active infected individuals started to rise again (green curve in Fig. 1). For these parameter values $\beta _i$, $p_i$, with $i = 1, 2, 3$, we estimated the parameter values $m_i$ (see “Methods” for details on the estimation of the parameters).

### Social opinion biased SAIRP model

The pandemic evolutions along past months, in different regions worldwide, demonstrated that the behavior of the population is of crucial influence. Same control policies, implemented in different regions, resulted in different outcomes. Even more, the same policies, implemented at different times, may produce different outcomes as the social state of opinion also changes with time.

We aim to incorporate the state of people’s opinion into the SAIRP model in order to analyze its influence. The process is divided into three steps. First, we calculate, from empirical data, the social network describing the social interactions for Portugal at two different moments of time (April and July 2020). With this information, we consider a simple opinion model that provides a probability distribution function that we interpret as the distribution of opinions to follow government policies (distributed from zero to one, zero meaning no intention to accept the policies and one total acceptance). As a final step, we introduce this probability distribution function into the SAIRP model by modulating the access to class *P*.

### Social opinion distributions

The details on the construction of the network, describing the social interactions, are explained in the “Methods” section. Just note that in both cases analyzed (April and July 2020) the network topology is quite different, reflecting a different social state. Each network is composed by a set of nodes (corresponding to different users or persons) and the connections with other nodes in the network. Both networks built, as described, constitute some kind of fingerprint of the social situation in Portugal at the specific periods of time considered.

**Figure 2:**
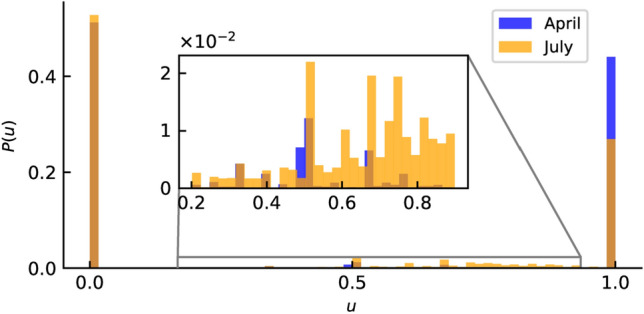
Probability distribution ($\mathbf {P(u)}$) **for each opinion** (${\mathbf {u}}$). The opinion ranges from zero to one, zero meaning no intention to follow the government policies while one means complete adhesion to this policy. The blue values correspond to the Portuguese situation in April 2020 while the yellow ones are for the situation in July 2020.

We use this network topology in order to incorporate a model of opinion. For that, we consider now that each node in our network is endowed with some dynamical equations, which allow to determine its state of opinion, combined with the information that it is coming through the network. The opinion dynamical equations are based on the logistic equations and they are fully described in the “Methods” section. The combined effect of the opinion model for each node, together with the influence of the information coming through the network, results in an opinion distribution function. The results are presented in Fig. 2. To each opinion in the *x*-axis it corresponds a probability to occur. In the two cases considered (April and July 2020) the opinion distribution appears very polarized, but in July we can detect a clear decrease in the intention to follow government imposed policies. This reflects the experience of the situation as it happened, during the worst of the pandemic (April) people were eager to follow any policy that helped reducing the impact of the disease, while in July more people changed the opinion and decide to oppose the restriction policies.

### SAIRP model with opinion distribution

Our aim now is to couple the previous SAIRP model with opinion distributions. For this purpose, instead of using a deterministic approach, we find more feasible a multi-agent based approach with stochastic dynamics, where a large number of individuals conform a mobility network and infected nodes can spread the disease through its connections with susceptible individuals^8^. The considered synthetic population is built according to the Watts–Strogatz model^23^, so it has small-world properties and high clustering. In particular, we considered a synthetic network with an average connectivity $\langle k \rangle =5$ and a probability of long range connections of $5\%$. Following the main idea of the SAIRP model, each node can be in one of the different compartments. Susceptible nodes can become asymptomatic by interactions with either asymptomatic or infected nodes, or become protected with probability $\phi p$, at each time step. At the same time, asymptomatic individuals are detected with probability $\nu$ and confirmed infected individuals can recover with probability $\mu$. Finally, protected individuals become susceptible again with probability $\omega$. The network is initialized with a discrete number of infected individuals and then these processes are evaluated until the dynamics of the disease become stationary.

We now introduce the opinion distributions through the protected *P* compartment. Considering the opinion probability distributions, *P*(*u*) (Fig. 2) for each node of the synthetic population we assign an opinion value drawn from *P*(*u*). Next, instead of having a fixed value for *p* and *m*, we consider that each node has its own probabilities of becoming protected and susceptible again, $p_i$ and $m_i$, and that these probabilities are given by the opinion value of the particular node. While we can directly identify $p_i$ with $u_i$, $m_i$ has to be related to the complementary of $u_i$: $\overline{u_i}=1-u_i$. Note that the meaning of the extreme values of the opinions are either to follow the directives and stay at home (if $u_i=1.0$) or not (if $u_i=0.0$). In this way, the opinion distributions overlap smoothly with the transition to the protected compartment. Finally, following the infection rate of the deterministic model, $\beta \cdot (1-p)$, we consider that the infection process occurs along the connection of an infected node *i* with a susceptible node *j* with probability $\beta \cdot (1-p_j)$. In this way, the infection process is also weighted by the opinion value of the susceptible node.

#### Remark

Although the values of $p_j$ are directly related to the $u_j$ values, their index *j* belong to completely different networks. On one hand, from the social network we extract the opinion distribution *P*(*u*), from which we build a new distribution *P*(*p*) with identical probabilities but applied to the epidemiological network (the one where we simulate the infective stochastic dynamics), assigning each node a value $p_j$.

**Figure 3:**
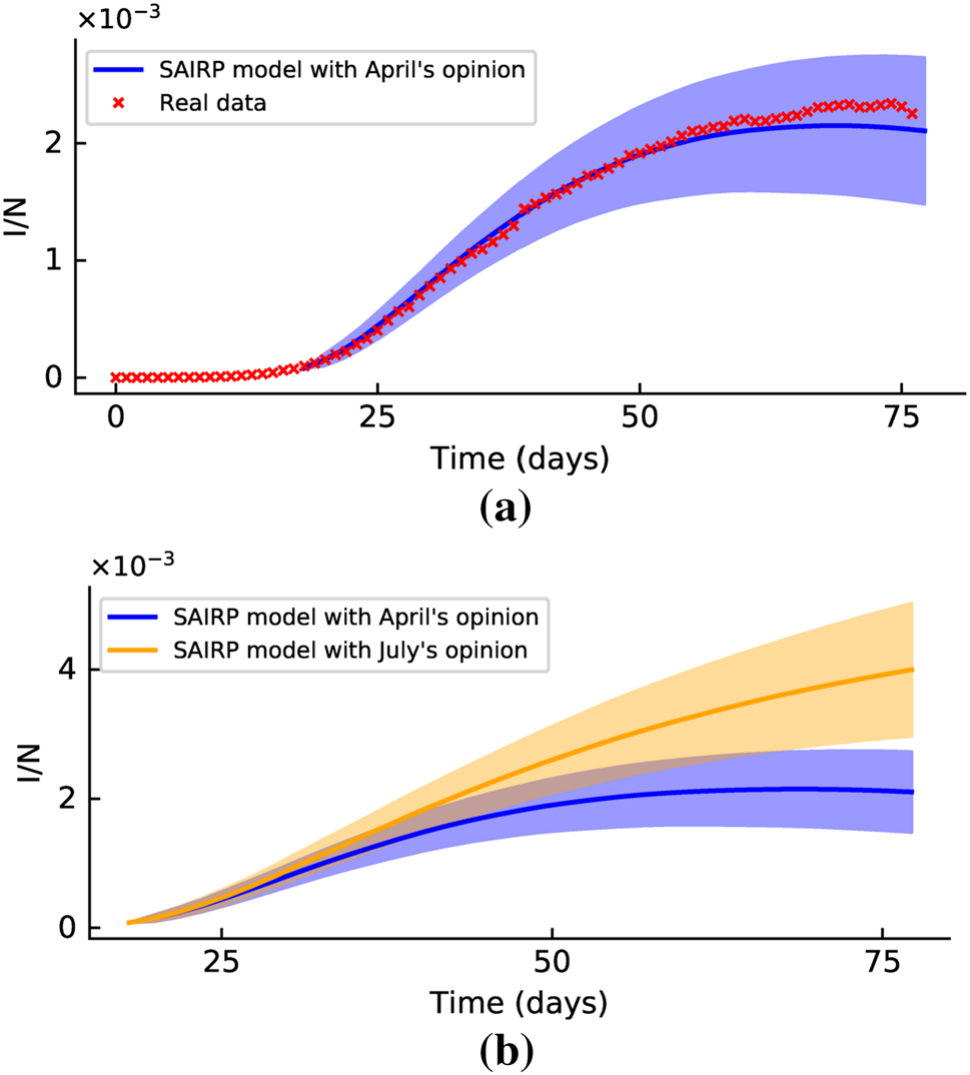
Evolution of the number of infected individuals (normalized by the total population) with time. (**a**) Red crosses correspond to the experimental recordings while the blue line is the fit of the SAIRP model with opinion. The bluish shadow marks the uncertainty of the model. (**b**) Blue line is the fit of the SAIRP model coupled with the opinion distribution, corresponding to April 2020, and the yellow line is the evolution of the model coupled with the state of social opinion as in July 2020.

The results of the SAIRP model with the opinion distributions included are presented in Fig. 3a. The red crosses mark the experimental observations until May 17 and the blue line is the fit to the SAIRP model with the opinion distribution. The model simulation was repeated 12000 times in order to gain statistical significance, i.e., the evolution of the number of infected individuals shown in Fig. 3 is consistent and does not depend on a limited number of realizations, but is rather generic as the average over a significantly large number of simulations. The parameters used for these simulations are in Table 4, in the “Methods” section.

In Fig. 3b, the results of the SAIRP model, coupled with the opinion distributions, are shown for the two situations considered. The blue line corresponds to the situation in April 2020. The yellow line shows a possible line of evolution of the pandemic in case the distribution of opinion is such as in July 2020 (the rest of the parameters were kept as in the blue curve). Note that the yellow line shows a much worse scenario and it is a direct conclusion of a change in the distribution of opinions.

### Optimal control

We obtain optimal control strategies that respect the following important constraints. (i) One needs to ensure that the number of hospitalized individuals with COVID-19 is such that the health system can respond to the other diseases in the population, in order that the mortality associated with other causes does not increase. (ii) It is important that the number of active infected individuals is always below a critical level. (iii) In order to keep the country “working”, there is always a percentage of the population that is susceptible to get infected. For instance, it is very important to keep schools open, in particular for children under 10/12 years old; there are always people that do not follow the rules imposed by the government; etc. Roughly speaking, our goal is to maximize the number of people that go back to “normal life” and minimize the number of active infected (and, consequently, the number of hospitalized and in ICUs), ensuring that the health system is never overloaded.

#### Hospitals and intensive care units occupancy beds by COVID-19

For the hospitalized individuals, the official data for the fraction of hospitalized individuals due to COVID-19, represented by *H*, with respect to the active infected individuals *I* is plotted in Supplementary Fig. 2 (a), *H*/*I*. We observe that after a first period, where all the active confirmed cases were hospitalized, the so-called *containment phase*, the percentage of active infected individuals that needs hospital treatment is always below 15%. Moreover, after the end of the emergency states (red dot in Supplementary Fig. 2), the percentage of active infected individuals that needs to be treated at hospitals is less or equal than 5% (the 15% and 5% are plotted with dotted blue lines in Supplementary Fig. 2).

For the percentage of active infected individuals that need to be in intensive care units (ICU), we observe that (see Supplementary Fig. 2 (b)) the proportion of active infected individuals that requires medical assistance in ICU is always below than $6\%$ and, moreover, after the end of the state of emergency the percentage of active infected individuals in the ICU is always below $1\%$.

#### Introduction of the control and its optimization

One of the main challenges, facing countries struck by the pandemic, is the reopening of the economy while preserving the health of the population without collapsing the public health system. It is very important to keep the schools open (remember that children under 10/12 years old are not obliged to use a mask in Portugal) and prevent the economy to sink. Thus, there is a minimum number of people that need to be susceptible to infection. But we also need to account that the population do not always follow the rules imposed by governments. We have developed tools to quantify this effect and include it into the equations. With this idea in mind, we investigate the use of optimal control theory to design strategies for this phase of the disease. The goal now is to maximize the number of people transferred from class *P* to the class *S* (that helps keeping the economy alive) and, simultaneously, minimize the number of active infected individuals and, consequently, the number of hospitalized and people needing ICU (in other words, ensuring that the health system is never overloaded). We want to impose that the number of active infected cases is always below 2/3 or 60% of the maximum value observed up to now ($I_{\max }$). This condition warrants that the health system does not collapse.

The fraction of protected individuals *P* that is transferred to susceptible *S*, is mathematically represented, in the *SAIRP* model, by the parameter *m*. The class of active infected individuals *I* is very sensitive to the change of the parameter *m* (Supplementary Fig. 3).

Taking into consideration the real official data of COVID-19 in Portugal^21^, let $I_{\max } = 2.5 \times 10^{-3}$ represent the maximum fraction of active infected cases observed in Portugal from March 2, 2020 until July 29, 2020. Note that for $m \geqslant 0.25$ the constraint $I(t) \leqslant 0.75 \times I_{\max }$ is not satisfied for the uncontrolled model (1). This means that the need of hospital beds and ICU beds can take vales such that the Health System can not respond, so we take the maximum value $I_{\max }$ as a reference point for the state constraints imposed on the optimal control problem, in order to ensure that in a future second epidemic wave the number of active infected cases remains below a certain percentage of this observed maximum value.

The parameter *m* in the *SAIRP* model, is replaced by a control function $u(\cdot )$. We formulate mathematically this optimal control problem and solve it (see “Methods”).

The control function *u* takes values between 0 and $u_{\max }$, with $u_{\max } \leqslant 1$. When the control *u* takes the value 0 there is no transfer of individuals from *P* to the class *S*; when *u* takes the value $u_{\max }$, then $u_{\max } \%$ of individuals in the class *P* are transferred to the class *S* at a rate *w* (see Table 2 in “Methods” for the meaning of parameter *w*).

We consider a time window of 120 days. In the Supplementary Information, we analyze with more detail the optimal control problem subject to $I \leqslant 2/3 \times I_{\max }$ and $u_{\max } \leqslant 0.95$ (see Supplementary Figs. 4–6 and Supplementary Table 1).

##### Remark

The optimal control problem under the state constraint $I \leqslant 2/3 \times I_{\max }$ is associated with a solution that implies a substantial and important difference on the number of hospital beds occupancy and in intensive care units with respect to the optimal control problem subject to the state constraint $I \leqslant 0.60 \times I_{\max }$. The choice of the constraints $I \leqslant 2/3 \times I_{\max }$ and $I \leqslant 0.60 \times I_{\max }$ comes from the mathematical numerical simulations carried out and the number of hospitals beds that the Portuguese Health System has available for COVID-19 assistance.

**Figure 4:**
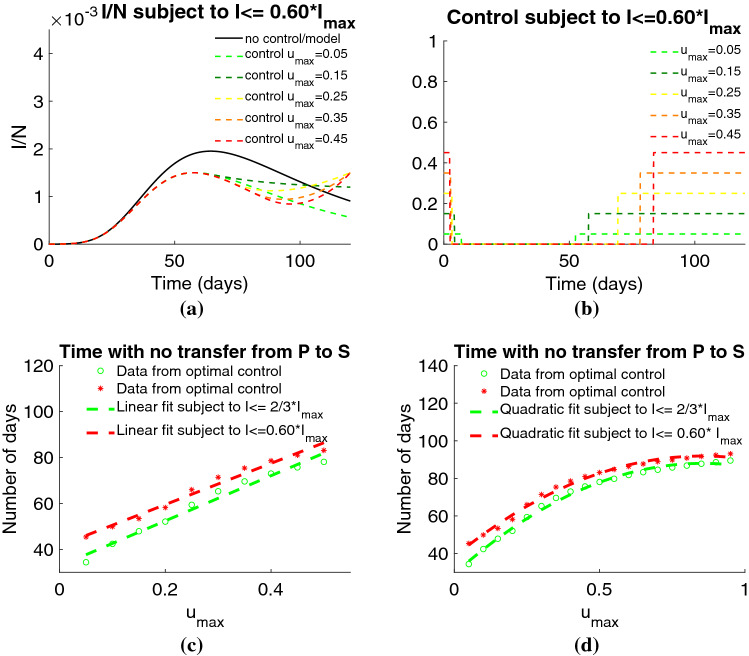
Active infected individuals: comparison of the solution of the SAIRP model with the optimal control problem. Linear and quadratic fit for the time where there is no transfer from *P* to *S*, in terms of $u_{\max }\in [0.05;0.95]$ under the constraints $I \leqslant 0.60 \times I_{\max }$ and $I \leqslant 2/3 \times I_{\max }$. (**a**) Fraction of active infected individuals. (**b**) Control *u* satisfying the constraint $I(t)\leqslant 0.60 \times I_{\max }$. (**c**) Linear fit for the time with no transfer from *P* to *S* for $0 < u_{\max } \leqslant 0.5$. (**d**) Quadratic fit for the time with no transfer from *P* to *S* for $0 < u_{\max } \leqslant 0.95$.

The controlled solution takes the maximum value $u_{\max }$ in a first period of time, followed by a period where there are no transfer of individuals from the class *P* to the class *S* and, at the final period of time, it takes the maximum value again (Fig. 4a,b). The case $u_{\max } > 0.5$ corresponds to a large number of days where there is no transfer of individuals from the class *P* to *S* (Fig. 4c,d and Supplementary Fig. 5).

The time with no transfer from *P* to *S* corresponds to a window of time where strict rules are imposed to the population, that can include home confinement, for example. This interval of time increases when the maximum value of the control $u_{\max }$ increases (see Supplementary Figs. 7 and 8). We are able to compute the absolute number of individuals that are released to the class *S* in terms of $u_{\max }$, which is a strictly increasing function of time (see Supplementary Fig. 9).

**Figure 5:**
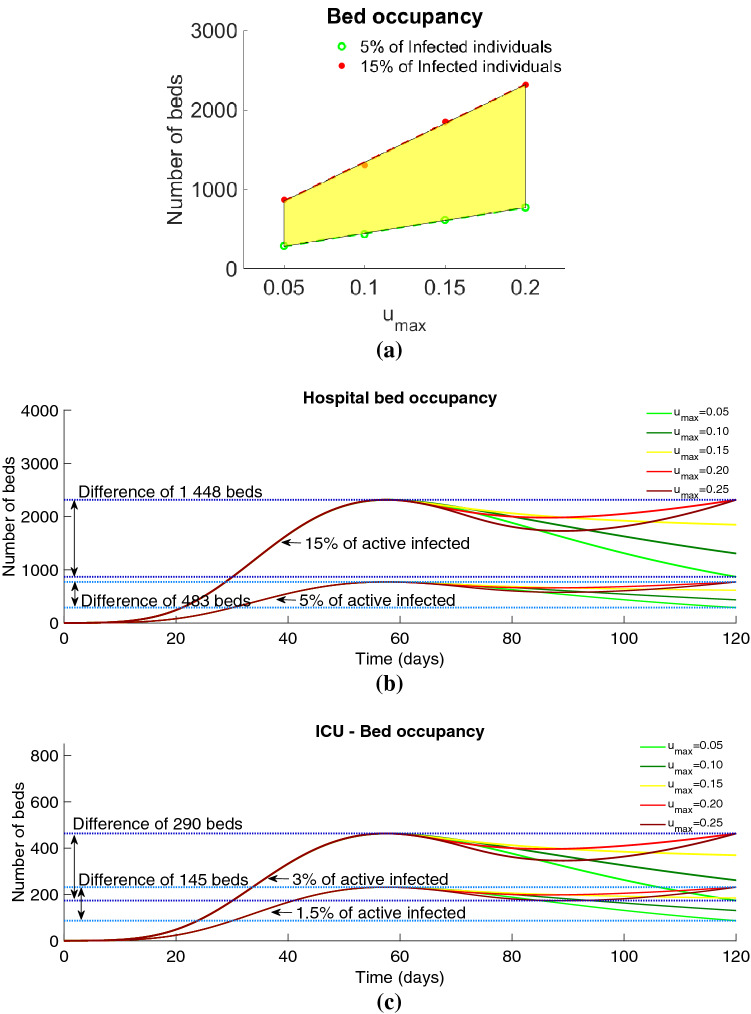
Number of hospital beds occupation for the optimal control solutions. (**a**) Number of hospital beds for $u_{\max }\in \{0.05, 0.10, 0.15, 0.20\}$ subject to $I(t)\leqslant 0.60 \times I_{\max }$ varying between 5% and 15% of the number of infected individuals. (**b**) Number of hospital beds for $u_{\max }\in \{0.05, 0.10, 0.15, 0.20, 0.25\}$ under the state constraint $I(t)\leqslant 0.60 \times I_{\max }$, representing between 5% and 15% of the number of active infected individuals. (**c**) ICU hospital bed occupancy for $u_{\max }\in \{0.05, 0.10, 0.15, 0.20, 0.25\}$ under the state constraint $I(t)\leqslant 0.60 \times I_{\max }$. The ICU beds occupation represents between 1.5% and 3% of the number of active infected individuals.

Without loss of generality, in what follows we consider $0 < u_{\max } \leqslant 0.25$, and analyze the hospital bed occupancy and ICU beds, due to COVID-19, associated to the optimal solutions that satisfy the constraint $I \leqslant 0.60 \times I_{\max }$ (see Fig. 5). For the bed occupancy due to COVID-19, we give information about the number of total beds needed in the cases where the percentage of active infected individuals that needs hospital care was between 5% and 15% (see Fig. 5a). This number is relatively small for the Portuguese capacities and will allow the medical assistance for non COVID-19 diseases.

Considering a range of values for maximum value of the percentage of *protected* individuals that is transferred to the *susceptible* between 0.05 and 0.25, that is $u_{max} \in \{ 0.05, 0.10, 0.15, 0.20, 0.25 \}$, the number of hospital beds needed to treat COVID-19 patients have a variation of 1448 beds, in the case when 15% of active infected individuals need medical assistance (see Fig. 5b). For the ICU bed occupancy, in the case where 3% of the active infected individuals require to be in ICU, the number of beds is presented in Fig. 5c and it may differ of 290 beds, when $u_{\max }$ varies from 0.05 to 0.25.

## Discussion

Portugal is a country that felt naturally isolated during most of the quarantine, so the data were not disturbed by spurious influences from other countries. Moreover, the disease was quite controlled at all times, the distribution of the population, as well as the distribution of social classes, is quite homogeneous countrywide and, thus, mathematical models are better suited to an analysis in a country like Portugal. Since the society behaves quite homogeneously across the country, we claim the social analysis here included to be quite relevant. To the best of our knowledge, this is the first work to investigate the reality of COVID-19 in Portugal and suggesting control measures coming from the mathematical theory of optimal control.

Optimal control theory is a branch of mathematics that offers a tool to tackle the problem of finding optimal strategies to stop the transmission of SARS-CoV-2. It is a powerful tool to design control strategies and act optimally on a given system. Based on reliable mathematical models for transmission mechanism of COVID-19, mathematical optimal control can thus help and assist the Public Health Authorities to understand, anticipate and mitigate the spread of the virus, and evaluate the potential effectiveness of specific prevention strategies.

A compartmental deterministic model describing the course of the epidemic, using data from Italy during the first 46 days (from February 20 through April 5, 2020), concluded that “restrictive social-distancing measures will need to be combined with widespread testing and contact tracing to end the ongoing COVID-19 pandemic”^6^. This has been implemented in Portugal. A stochastic microsimulation agent-based model of the SARS-CoV-2 epidemic for France concluded that “lockdown is effective in containing the viral spread, once lifted, regardless of duration, but it would be unlikely to prevent a rebound.” The model calibrated well, based on a visually good fit between observed and model-predicted daily ICU admissions, ICU-bed occupancy, daily mortality and cumulative mortality^8^. Our model goes further; it does the fit of active infected individuals and, based on that, estimates the number of hospitalized individuals with COVID-19 and the ones that are in ICU. A projection of the SARS-CoV-2 transmission dynamics through a postpandemic period, has been carried out with the help of a SEIR model with two strains^9^. For that, time-series data from USA has been used to calibrate the SARS-CoV-2 transmission model^9^. They concluded that a prolonged or intermittent social distancing may be necessary into 2022, with additional interventions, including expanded critical care capacity and an effective therapeutic, for the acquisition of herd immunity to be possible^9^. Instead of recommending the expansion of care capacity, here we propose measures that maintain the number of active cases in a low level. Teslya et al.^24^, suggest that information dissemination about COVID-19, which causes individual adoption of hand-washing, mask-wearing, and social distancing, can be an effective strategy to mitigate and delay the epidemic, stressing the importance of disease awareness in controlling the ongoing epidemic and recommending, in addition to policies on social distancing, that governments and public health institutions mobilize people to adopt self-imposed measures with proven efficacy in order to successfully tackle COVID-19. This was the case in Portugal. The Portuguese experience, which prevented the rupture of the national health system, shows that health literacy should be a central objective at reach. Before political power closed schools and other institutions, the community anticipated and it took preventive measures. In our study, more than that, we use optimal control and network theories with social opinion to enrich such efforts. Although many other mathematical models have been already proposed for COVID-19, the model we introduce here allows to represent, with a good fit, the fraction of active infected individuals in Portugal, for more than 150 days, and provides an interesting balance between much more complex models, with several more compartments, and the too much simplistic SIR/SEIR models. Furthermore, in this work we do not simply study the sensitivity of the model to the change of the fraction of individuals that is in the protected class and goes back to the susceptible, which can be done by changing some parameter values, but we propose optimal control solutions.

In many countries, Portugal included, the so-called non-pharmaceutical interventions (NPIs) were taken since the first confirmed case. Therefore, our mathematical model considers a class of individuals that practice, in an effective way, the NPIs measures and, therefore, is *protected* from the virus. Based on recent studies^19,20^, we assume that the individuals that follow NPIs measures are protected from infection of SARS-CoV-2. It is important to keep people in the class of protected/prevented due to the existing risk of transmission of the infection by asymptomatic infected individuals^25^.

We propose a *SAIRP* mathematical model, that represents the transmission dynamics of SARS-CoV-2 in a homogeneously mixing constant population. The *SAIRP* model fits the confirmed active infected individuals in Portugal, from the first confirmed case, on March 2, 2020, until July 29, 2020, using real data from Portuguese National Authorities^21^. The new model considers a class of individuals that we call *protected/prevented*, representing the fraction of individuals that is under effective protective measures, preventing the spread of SARS-CoV-2. In a first phase, from March 14 until May 02, 2020, this class represented all the individuals that were in confinement, due to closed schools, layoff, etc. After the three states of emergency implemented in Portugal, the confinement measures started to be raised but, simultaneously, other prevention measures were recommended by the Government, such as the use of mask, that became mandatory in closed spaces. All the individuals that practice, in a effective way, all NPIs, are considered to belong to class *P*. The social opinion network implemented shows how the Portuguese population has followed the health authorities policies and recommendations: social distance, use of mask, avoid of celebrations, etc. In practice, this can be related to the partial maintenance of the population in the class *P* of the *SAIRP* model. However, there is always a significant percentage of the population that does not follow, in an effective way, the official recommendations. Moreover, there are groups in the population that are crucial to a “normal life” and cannot avoid close physical and unprotected contacts, such as children in kindergartens and primary schools. With this background, we formulate an optimal control problem, where the control represents the percentage of protected/prevented individuals that are transferred to the susceptible class, that is, is not under protective measures. The goal to consider such optimal control problem is to find the optimal strategy to transfer individuals from *protected/prevented* class to the class of *susceptible*, with minimal active infected individuals and always below a specific threshold that maintains the number of hospitalized individuals due to COVID-19 and hospitalized in intensive care units, below the level that the National Health Service is able to answer while keeping the other “usual” medical services working normally. This is also connected with the political and social interest of keeping the economy open and “active”. We provide the mathematical optimal control solutions for different scenarios on the fraction of protected individuals that is transferred to the susceptible class and also for different threshold levels.

We conclude with some words explaining why we believe optimal control has an important role in helping to prevent COVID-19 dissemination, and also pointing out some possible future research directions. In general, the response to chronic health problems has been impaired, both because the resources were largely allocated to COVID-19 or because the population was afraid to go to the hospitals and many surgeries and consultations remain to be made. Many institutions have organized what has been called in Portugal “home hospitalization”, which served to mitigate many problems that would remain unanswered. Hospital teams, multidisciplinary teams, systematically moved to the homes of patients and sought care in their environment, avoiding nosocomial infections and also the occupation of beds. This experience was evaluated as very positive by the Portuguese population. Most probably, this coronavirus will remain in the communities for many years, so the changes we see in health services and in people’s habits have to go on over time. Actions as simple as hand washing, space hygiene, social distance and use of masks in closed spaces, should be incorporated into education for health. The containment measures, which should be necessary when outbreaks arise, must be rigorously studied and worked with families. Confinement cannot mean social isolation and should be worked out according to each family reality. The latest data shows that European countries are already at the limit in terms of reinforcements to NHS budgets. Changing many hospital practices, such as cleanliness and hygiene, food services, relationship between emergencies and hospitalization, support for clinical training of health professionals, etc., can help to rationalize resources and prevent infections to other users, especially in autumn and winter, where different forms of flu and pneumonia burden institutions. At this moment we do not include such “social” corrections in the optimal control part, but it would be interesting to consider them in future work.

## Methods

### Mathematical epidemiological model

The SAIRP model (1) subdivides human population into five mutually-exclusive compartments (see Table 1 and Supplementary Fig. 1), representing the dynamical evolution of the population in each compartment over a fixed interval of time.

**Table 1:** Description of the population model compartments.

Population compartment	Description
*S*	Susceptible
*A*	Asymptomatic
*I*	Confirmed/active infected
*R*	Recovered/removed (includes deaths by COVID-19)
*P*	Protected/prevented

The susceptible individuals become infected by SARS-CoV-2 by contact with infected asymptomatic *A* and active infected individuals *I*. The rate of infection is given by $\beta \left( \theta A(t) + I(t) \right)$, where $\beta$ is the infection transmission rate of active infected individuals *I* and $\theta$ represents a modification parameter for the infectiousness of the asymptomatic infected individuals (*A*). A fraction *p*, with $0< p < 1$, is protected from infection by SARS-CoV-2, due to an effective implementation of non-pharmaceutical interventions (NPIs) and is transferred to the class *P*, at a rate $\phi$. However, individuals in the class *P* are not immune to infection and a fraction *m* can become susceptible again at a rate *w*. For the sake of simplification, we denote $\omega = w m$. A fraction *q* of asymptomatic infected individuals *A* develop symptoms and are detected, at a rate *v*, being transferred to the class *I*. We use the notation $\nu = v q$. Active infected individuals *I* exit this class either by recovery from the disease or by COVID-19 induced death, being transferred to the class of removed/recovery *R*, at a rate $\delta$ (see Table 2).

**Table 2:** Description of the parameters of model (1).

Parameter/	Description
$\beta$	Infection transmission rate
$\theta$	Modification parameter
*p*	Fraction of susceptible *S* transferred to protected class *P*
$\phi$	Transition rate of susceptible *S* to protected class *P*
$$\omega = w m$$	
*w*	Transition rate of protected *P* to susceptible *S*
*m*	Fraction of protected *P* transferred to susceptible *S*
$$\nu = v q$$	
*v*	Transition rate of asymptomatic *A* to active/confirmed infected *I*
*q*	Fraction of asymptomatic *A* infected individuals
$\delta$	Transition rate from active/confirmed infected *I* to removed/recovered *R*

The previous assumptions are described by the following system of five ordinary differential equations:

1 $$\begin{aligned} {\left\{ \begin{array}{ll} {\dot{S}}(t) = - \beta (1-p) \left( \theta A(t) + I(t) \right) S(t) - \phi p S(t) + \omega P(t) ,\\ {\dot{A}}(t) = \beta (1-p) \left( \theta A(t) + I(t) \right) S(t) - \nu A(t) , \\ {\dot{I}}(t) = \nu A(t) - \delta I(t) ,\\ {\dot{R}}(t) = \delta I(t), \\ {\dot{P}}(t) = \phi p S(t) - \omega P(t) . \end{array}\right. } \end{aligned}$$

#### Remark

The testing rate in Portugal, as in many other countries, has been increasing since the beginning of the pandemic. However, in our model we do not consider the impact of the testing rate on the detection of infected cases. This is due to the fact that in Portugal only suspected individuals that had a close contact, without mask protection, or individuals with COVID-19 symptoms, are tested.

#### Remark

In our model (1), individuals from compartment *A* move to compartment *I*. Given testing frequencies and reliability, we adjust the infection rate and consider a proportion of detection. The parameter *q* is used to obtain the proportion of *A* moving to *I*. In a general framework, a fraction $(1-q)$ of asymptomatic individuals *A* should be transferred to the compartment *R*. However, in this work we are based on the official data provided by The Portuguese Health Authorities and our aim is to propose a mathematical model that fits well the reality described by the daily reports data, more specifically the curve of the active infected individuals by COVID-19 in Portugal and, sub-sequentially, the fraction of active individuals that are hospitalized and in intensive care units. Using official data, only the individuals that were confirmed to be infected by testing (the ones that are represented by the class *I*) may be transferred to the class *R*. Therefore, since the asymptomatic are not counted in the official data, it is not possible (in this model) to count them as recovered after a certain number of days.

Let us define the total population *N* by $N(t) = S(t) + A(t) + I(t) + R(t) + P(t)$. Taking the derivative of *N*(*t*), it follows from (1) that ${\dot{N}}(t) = 0$, that is, *N* is constant over time. Without loss of generality, we normalize the system so that $N = 1$. All parameters of the model are non-negative and, given non-negative initial conditions $(S_0, A_0, I_0, R_0, P_0) = \left( S(0), A(0), I(0), R(0), P(0)\right)$, the solutions of system (1) are non-negative and satisfy $S(t)+A(t)+I(t)+R(t)+P(t) = 1$ for all time $t \in [0, t_f]$. With this conservation law, the model (1) can be simplified to 4 equations, the cumulative number of removed/recovered individuals *R*(*t*) being given, for each $t \geqslant 0$, by

2 $$\begin{aligned} R(t) = R(0) + \delta \int _{0}^{t} I(s) \, ds \, . \end{aligned}$$

Therefore, we consider the following *SAIP* simplified model for the optimal control problem formulation:

3 $$\begin{aligned} {\left\{ \begin{array}{ll} {\dot{S}}(t) = - \beta (1-p) \left( \theta A(t) + I(t) \right) S(t) - \phi p S(t) + \omega P(t),\\ {\dot{A}}(t) = \beta (1-p) \left( \theta A(t) + I(t) \right) S(t) - \nu A(t) , \\ {\dot{I}}(t) = \nu A(t) - \delta I(t) ,\\ {\dot{P}}(t) = \phi p S(t) - \omega P(t) . \end{array}\right. } \end{aligned}$$

#### Remark

In our model we are taking into account the infectiousness of fully asymptomatic patients *A*. In concrete, the transmission incidence is given by the term $\beta (1-p) (\theta A(t) + I(t))$.

The disease free equilibrium $\Sigma _0$ of model (3) is given by

4 $$\begin{aligned} \Sigma _0 = \left\{ S=\frac{\omega }{\phi \,p+\omega }, \, A=0, \, I=0, \, P=\frac{\phi \,p}{\phi \,p+\omega } \right\} \end{aligned}$$

with $S + P = 1$. Following the approach of Driessche and Watmough^26^, the basic reproduction number $R_0$ is given by the spectral radius of $FV^{-1}$, where the matrices *F*, *V* and $FV^{-1}$ are given by

$$\begin{aligned} F= & {} \begin{pmatrix} -S\beta (p-1)\theta &{} -\beta (p-1)S \\ 0 &{} 0 \end{pmatrix} ,\qquad V = \begin{pmatrix} \nu &{} 0 \\ -\nu &{} \delta \end{pmatrix} \, ,\\ FV^{-1}= & {} \begin{pmatrix} -{\frac{\beta (p-1)\theta \omega }{(\phi p+\omega )\nu }} -{\frac{\beta (p-1) \omega }{(\phi p+\omega ) \delta }}\quad &{} -{\frac{\beta (p-1) \omega }{( \phi p+\omega ) \delta }} \\ 0 &{} 0 \end{pmatrix}, \end{aligned}$$

that is,

5 $$\begin{aligned} R_0 = \frac{\beta (1-p)\, \omega \, (\theta \delta +\nu )}{(\phi p+\omega )\, \nu \,\delta } \, . \end{aligned}$$

### Parameter values and estimation from Portuguese COVID-19 data

We consider official data, where daily reports are available with the information about total (cumulative) confirmed infected cases, total recovered, and total deaths by COVID-19 in Portugal, and also information about the number of hospitalized individuals and in intensive care due to COVID-19 disease^21^.

**Table 3:** Initial conditions and parameter values for Portugal from March 2, 2020 to June 19, 2020. Contrast with Fig. 1. The parameters $$\beta _1$$, $$\beta _2$$ and $$\beta _3$$, and $$m_1$$, $$m_2$$ and $$m_3$$ were estimated using the Matlab function lsqcurvefit for $$t \in [0, 77]$$ and $$t \in [100, 150]$$ days, respectively. From March 2 to May 17, 2020 (77 days): $$t \in [0, 77]$$ – $$\beta _1 = 1.492$$, $$m_1 = 0.059$$ and $$p_1 = 0.675$$. From May 17 to June 9, 2020 (23 days): $$t \in [77, 100]$$ – $$\beta _2 = 0.25$$, $$m_2 = 0.058$$ and $$p_2 = 0.4$$. From June 9 to July 29, 2020 (50 days): $$t \in [100, 150]$$ – $$\beta _3 = 1.91$$, $$m_3 = 0.043 $$ and $$p_3 = 0.4$$.

Parameter/Initial condition	Value	Reference
$\beta _1$	1.492	Estimated
$\beta _2$	0.25	Estimated
$\beta _3$	1.91	Estimated
$\theta$	1	^27^
$p_1$	0.675	^21,22^
$p_2$	0.55	
$p_3$	0.40	
$\phi$	$1/12 \, day^{-1}$	^22,32^
$$\omega = w m$$		
*w*	$1/45 \, day^{-1}$	^22^
$m_1$	0.059	Estimated
$m_2$	0.058	Estimated
$m_3$	0.043	Estimated
$$\nu = v q$$		
*v*	$1 \, day^{-1}$	
*q*	0.15	^28–30^
$\delta$	$1/30 \, day^{-1}$	^31^
$N = S_0 + A_0 + I_0 + R_0 + P_0$	10295909	^34^
$S_0$	10295894/*N*	^21^
$I_0$	2/*N*	^21^
$A_0$	(2/0.15)/*N*	^21^
$R_0$	0	^21^
$P_0$	0	^21^

We assume $\theta = 1$ for the current (up to the date) best estimate for the infectiousness of asymptomatic individuals relative to symptomatic individuals^27^. For the fraction of asymptomatic *A* infected individuals, we consider $q = 0.15$^28–30^. The parameter *w* takes the value $w=1/45 \, day^{-1}$, corresponding to the 3 emergency states (duration 45 days)^22^. The value of the parameter $\delta$, representing the recovery time of confirmed active infected individuals *I*(*t*) (with negative test)/removed (by death), is assumed to be $\delta = 1/30 \, \text {days}^{-1}$, considering that here might be a delay on the publication of real data^31^. The parameters $\beta _1$, $\beta _3$, $m_1$ and $m_3$ were estimated using the Matlab function lsqcurvefit for $t \in [100, 150]$ days, respectively. From March 2 to May 17, 2020 (77 days): $t \in [0, 77]$ – $\beta _1 = 1.492$, $m_1 = 0.059$, and $p_1 = 0.675$. From May 17 to June 9, 2020 (23 days): $t \in [77, 100]$ – $\beta _2 = 0.25$, $m_2 = 0.058$ and $p_2 = 0.4$. From June 9 to July 29, 2020 (50 days): $t \in [100, 150]$ – $\beta _3 = 1.91$, $m_3 = 0.043$, and $p_3 = 0.4$. The fraction $0< p_1 < 1$, for $t \in [0, 77]$, is assumed to take the value $p_1=0,675$, representing the population affected by the confinement of policies^21,22^. For $t \in [77, 100]$, we assume a decrease of the fraction of *protected* individuals to $p_2=0.55$. For $t \in [100, 150]$, we assume $p_3 = 0.44$, based on a gradual transfer of individuals from the class *P* to the class *S*. The transfer of individuals from *S* to *P* started on March 14, 2020^22,32^, thus we take $\phi = 1/12 \, day^{-1}$.

### Building the social network

In order to generate the social network, we use data collected from the micro-blogging website Twitter. With aid of the Python package GetOldTweets3^33^, we were able to download a collection of several tweets (posts of 244 characters) attending to participation in a given hashtag. Merging a handful of different hashtags, we obtained a significant sample of users who are interacting between themselves, either exchanging information with replies or spreading it via what is called a “re-tweet”. The more hashtags we use, the more realistic is the reconstruction of the social network in regards to the actual situation of the Portugal Twitter network. In mathematical terms, we build a complex neiork where users lie in the nodes and the directed edges represent the interactions between users. We are mainly interested on the structure of the interactions rather than the topic of the information, and thus we discard everything related to the personal information of the users and the content of the tweets.

Most real world networks are changing in time, either by changes on the connectivity pattern or either by growth and continuous addition of new nodes. This is a key feature of the so called scale-free complex network^35^, and it is a feature shared by the social network Twitter^36–38^. Thus, the structure of the network can drastically change from one month to another, and so it is important to take this point into account when building the network. In our data, this was accomplished by a feature of the used package, which allows to filter the search by date. In fact, here we were also interested in comparing the behavior of the social network during April, when the quarantine was imposed, and the social network during July, when the social distancing measures relaxed. The connectivity distributions for both networks are shown in Supplementary Fig. 10. In both cases, the topology corresponds with that of a *scale free network* but with rather different exponents, $\gamma _{April}=2.11$ and $\gamma _{July}=1.82$. The significantly different exponents demonstrate the different internal dynamics in both cases, which are reflected in the opinion distributions.

### Opinion model

The network topology obtained was endowed with a dynamical opinion set of equations for each node (actual person) that, combined with the information coming through the network connections, allowed it to produce an opinion. We considered a simple opinion model based on the logistic equation^39^ but that has proved to be of use in other contexts^40–42^. The equations describing each node *i*, $i=1, \ldots , N$, are^42^:

6 $$\begin{aligned} \frac{d u_i}{d t} = f(u_i) + d \frac{1}{k_i} \sum _{j=1}^{N} L_{ij} u_{j}, \end{aligned}$$

where $u_i$ is the opinion of node *i* that ranges from zero to one. The nonlinearity $f(u_i)$ is given by the following equation:

7 $$\begin{aligned} f(u)=u \left( A (1 - u/B) + g(1-u) \right) . \end{aligned}$$

Each of the nodes *i* obeys the internal dynamic given by $f(u_i)$ while being coupled with the rest of the nodes with a strength $d/k_i$, where *d* is a diffusive constant and $k_i$ is the connectivity degree for node *i* (number of nodes each node is interacting with). Note that this is a directed non-symmetrical network where $k_i$ means that node *i* is following the tweets from $k_i$ nodes and, thus, it is being influenced by those nodes in its final opinion. The Laplacian matrix $L_{ij}$ is the operator for the diffusion in the discrete space, $i=1,\ldots ,N$. We can obtain the Laplacian matrix from the connections established within the network as $L_{ij}=A_{ij}-\delta _{ij} k_i$, being $A_{ij}$ the adjacency matrix:

8 $$\begin{aligned} A_{ij}= {\left\{ \begin{array}{ll} 1\quad \text {if} \quad i,j \text { are connected,} \\ 0\quad \text {if} \quad i,j \text { are not connected}. \end{array}\right. } \end{aligned}$$

Now, we proceeded as follows. We considered that all the accounts (nodes in our network) were in their stable fixed point with a $10\%$ of random noise. Then a subset of the nodes was forced to acquire a different opinion, $u_i=1$ with a $10\%$ of random noise and we let the system to evolve following the above dynamical equations. The influence of the network made some of the nodes to shift their opinion to values closer to 1 that, in the context of this simplified opinion model, means that those nodes shifted their opinion to values closer to those leading the shift in opinion. This process was repeated in order to gain statistical significance and, as a result, it provided the probability distribution of nodes eager to change the opinion and adhere to the new politics. The parameter values used were $A=0.00001$, $B=0.1$, $g=0.001$ and $d=1.0$.

### Parameter values for the SAIRP model with opinion distributions

The parameters used and the initial conditions are summarized in Table 4. Once the opinion distribution was included into the SAIRP model, the parameters were slightly adjusted to be able to continue describing accurately the experimental situation. In fact, moving from an only-time-dependent-model to the network type model we consider in this section implies that the whole dynamic of the system is speeded up as now each node has the capability to trigger the epidemic wave. In order to compensate this effect, a rescaling of the parameters controlling the temporal scale in the system, namely $\delta$, $\phi$ and *w*, is necessary. An estimated rescaling factor of 1.85 leaves the modified parameters shown in Table 4. With the opinion distribution included into the SAIRP model, the infection transmission rate also needs to be adjusted in order to continue describing accurately the experimental situation.

**Table 4:** Initial conditions and parameter values for Portugal from March 2, 2020 to May 17, 2020 for the SAIRP model, modified by the opinion distributions.

Parameter/Initial condition	Value
$\beta$	0.2
$\phi$	$1/6.486 \, day^{-1}$
*w*	$1/24.32 \, day^{-1}$
*v*	$1 \, day^{-1}$
*q*	0.15
$\delta$	$1/16.216 \, day^{-1}$
$N = S_0 + A_0 + I_0 + R_0 + P_0$	25000
$$S_0$$	$$ (N-2-2/0.15)/N$$
$I_0$	2/*N*
$A_0$	(2/0.15)/*N*
$R_0$	0
$P_0$	0

### Optimal control problem

The goal is to find the optimal strategy for letting people to go out from class *P* to the class *S* and, at the same time, minimize the number of active infected while keeping the class of active infected individuals below a safe maximum value.

The control $u(\cdot )$ represents the fraction of individuals in class *P* of *protected* that is transferred to the class *S*. The control *u* is introduced into the *SAIP* model in the following way:

9 $$\begin{aligned} {\left\{ \begin{array}{ll} {\dot{S}}(t) = - \beta (1-p) \left( \theta A(t) + I(t) \right) S(t) - \phi p S(t) + w u(t) P(t) ,\\ {\dot{A}}(t) = \beta (1-p) \left( \theta A(t) + I(t) \right) S(t) - \nu A(t) , \\ {\dot{I}}(t) = \nu A(t) - \delta I(t) ,\\ {\dot{P}}(t) = \phi p S(t) - w u(t) P(t) . \end{array}\right. } \end{aligned}$$

The control must satisfy the following constraints: $0 \leqslant u(t) \leqslant u_{\max }$ with $u_{\max } \leqslant 1$. In other words, the solutions of the problem must belong to the following set of admissible control functions:

10 $$\begin{aligned} \Theta = \left\{ u, \; u \in L^1 \left( [0, t_f], \mathbb {R} \right) \, | \, 0 \leqslant u(t) \leqslant u_{\max } \, \;\; \forall \; t \in [0, t_f] \right\} \, . \end{aligned}$$

Mathematically, the main goal consists to minimize the cost functional

11 $$\begin{aligned} J(u) = \int _{0}^{t_f} k_1 I(t) - k_2 \, u(t) \, dt \, , \end{aligned}$$

representing the fact that we want to minimize the fraction of infected individuals *I* and, simultaneously, maximize the intensity of letting people from class *P* go back to class *S*. The constants $k_i$, $i=1, 2$, represent the weights associated to the class *I* and control *u*. Moreover, the solutions of the optimal control problem must satisfy the following state constraints: $I(t) \leqslant \zeta$ with $\zeta = 0.6 \times I_{\max }$ and $\zeta = 2/3 \times I_{\max }$.

For the numerical simulations, we considered $k_1 = 100$, $k_2 = 1$ and $t_f = 120$ days. We also considered $(\beta , \delta ) = (1.464, 1/30)$, $m = 0.09$, $p = 0.675$, and all the other parameters from Table 3. Numerically, we discretized the optimal control problem to a nonlinear programming problem, using the Applied Modeling Programming Language (AMPL)^43^. After that, the AMPL problem was linked to the optimization solver IPOPT^44,45^. The discretization was performed with $n = 1500$ grid points using the trapezoidal rule as the integration method.

## Supplementary Information

Supplementary Information.

## Data Availability

All of the data are publicly available and were extracted from https://covid19.min-saude.pt/relatorio-de-situacao/.

## Code availability

The code is available from the authors on request.
