# Supplementary material for: Optimal control of the COVID-19 pandemic: controlled sanitary deconfinement in Portugal
Source: Sci Rep. 2021 Feb 10;11:3451. doi: 10.1038/s41598-021-83075-6 (PMC7876047; doi:10.1038/s41598-021-83075-6)
Supplement: Supplementary file 1 — Supplementary Information. [file 41598_2021_83075_MOESM1_ESM.pdf]

# Optimal control of the COVID-19 pandemic: controlled sanitary deconfinement in Portugal


Cristiana J. Silva[*,1] cjoaosilva@ua.pt
Carla Cruz[1] carla.cruz@ua.pt
Delfim F. M. Torres[1] delfim@ua.pt
Alberto P. Muñuzuri[2] alberto.perez.munuzuri@usc.es
Alejandro Carballosa[2] ac.carballosa@gmail.com
Iván Area[3] area@uvigo.gal
Juan J. Nieto[4] juanjose.nieto.roig@usc.es
Rui Fonseca-Pinto[5] rui.pinto@ipleiria.pt
Rui Passadouro[5,6] rmfonseca@arscentro.min-saude.pt
Estevão Soares dos Santos[6] essantos3@arscentro.min-saude.pt
Wilson Abreu[7] wjabreu@esenf.pt
Jorge Mira[*,8] jorge.mira@usc.es

[1]Center for Research and Development in Mathematics and Applications (CIDMA), Department of Mathematics, University of Aveiro, 3810-193 Aveiro, Portugal
[2]Institute CRETUS, Group of Nonlinear Physics, Department of Physics, Universidade de Santiago de Compostela, 15782 Santiago de Compostela, Spain
[3]Departamento de Matemática Aplicada II, E. E. Aeronáutica e do Espazo, Campus de Ourense, Universidade de Vigo, 32004 Ourense, Spain
[4]Instituto de Matemáticas, Universidade de Santiago de Compostela, 15782 Santiago de Compostela, Spain
[5]Center for Innovative Care and Health Technology (ciTechCare), Polytechnic of Leiria, Portugal
[6]ACES Pinhal Litoral – ARS Centro, Portugal
[7]School of Nursing & Research Centre "Centre for Health Technology and Services Research / ESEP-CINTESIS", Porto, Portugal
[8]Departamento de Física Aplicada, Universidade de Santiago de Compostela, 15782 Santiago de Compostela, Spain

# Supplementary Figures

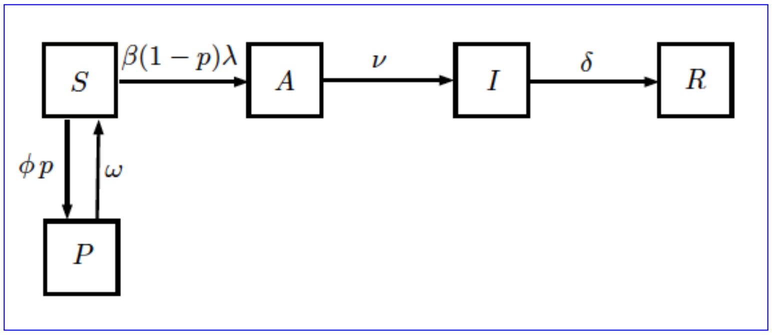


**Supplementary Figure 1: Diagram of the SAIRP model for the transmission dynamics of SARS-CoV-2 in a homogeneous population.** The population is subdivided into five compartments depending on the state of infection and disease of the individuals: $S$, susceptible (uninfected and not immune); $A$, infected but asymptomatic (undetected); $I$, active infected (symptomatic and detected/confirmed); $R$, removed (recovered and deaths by COVID-19); $P$, *protected/prevented* (not infected, not immune, but that are under protective measures).

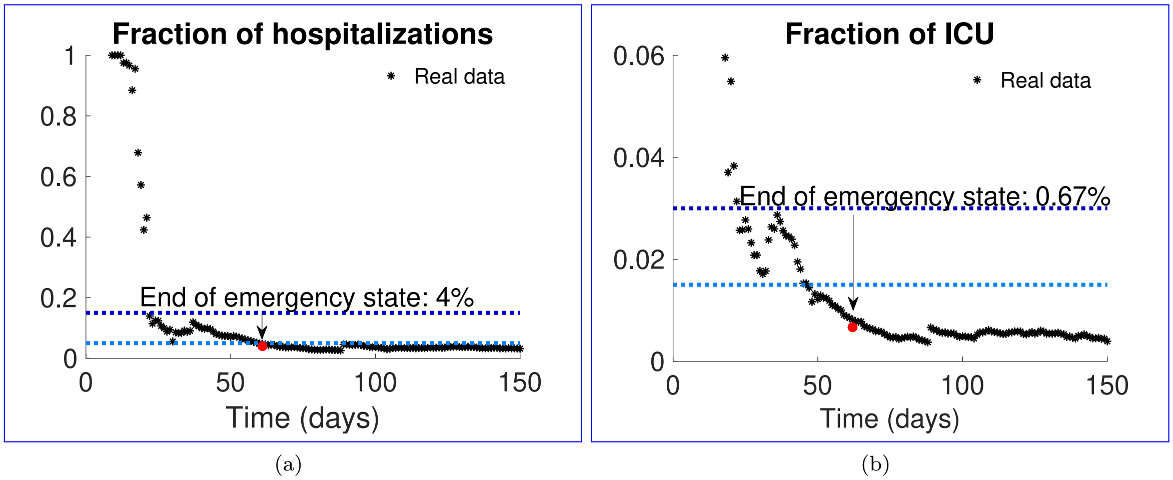


**Supplementary Figure 2: Official real data, from March 02 to July 29, for the fraction of hospitalized individuals and in ICU due to COVID-19, with respect to the confirmed/active infected individuals.** (a) Fraction of hospitalized individuals due to COVID-19 with respect to the number of active infected individuals, $H/I$. (b) Fraction of intensive care units (ICU) hospitalized individuals due to COVID-19 with respect to the number of active infected individuals, $ICU/I$.

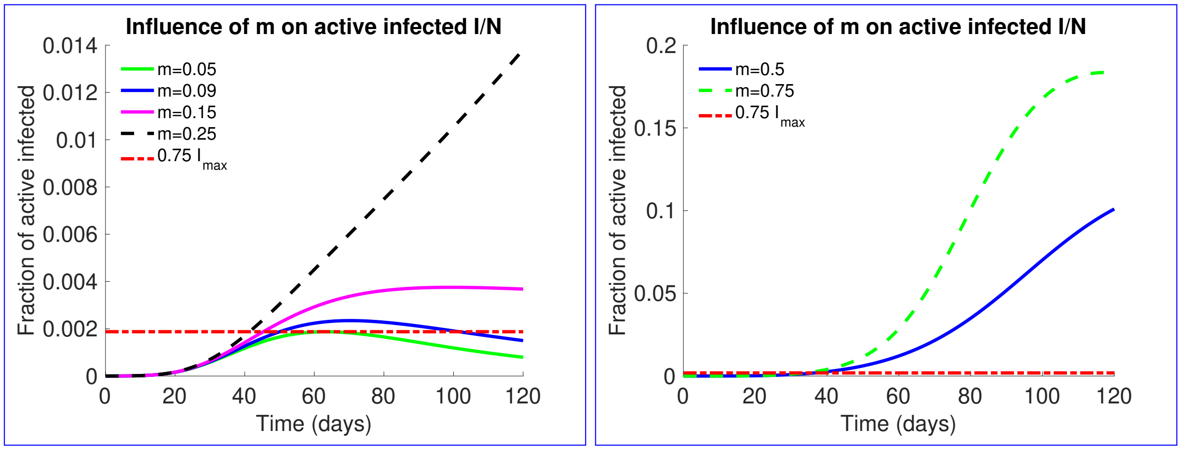


(a) (b)

**Supplementary Figure** 3: **Sensitivity of class I with respect to parameter m.** Fraction of active infected individuals $I$ for: (a) $m \in \{0.05, 0.09, 0.15, 0.25\}$, the dotted red line marks the level $0.75 \times I_{\max}$ that represents approximately 75% of the maximum fraction of active infected cases observed in Portugal (up to July 29, 2020); (b) $m \in \{0.5, 0.75\}$, the dotted red line marks the level $0.75 \times I_{\max}$ that represents approximately 75% of the maximum fraction of active infected cases observed in Portugal. We consider the fixed parameters $(\beta, p) = (1.464, 0.675)$ and all the other parameters from Table 3 in Methods.

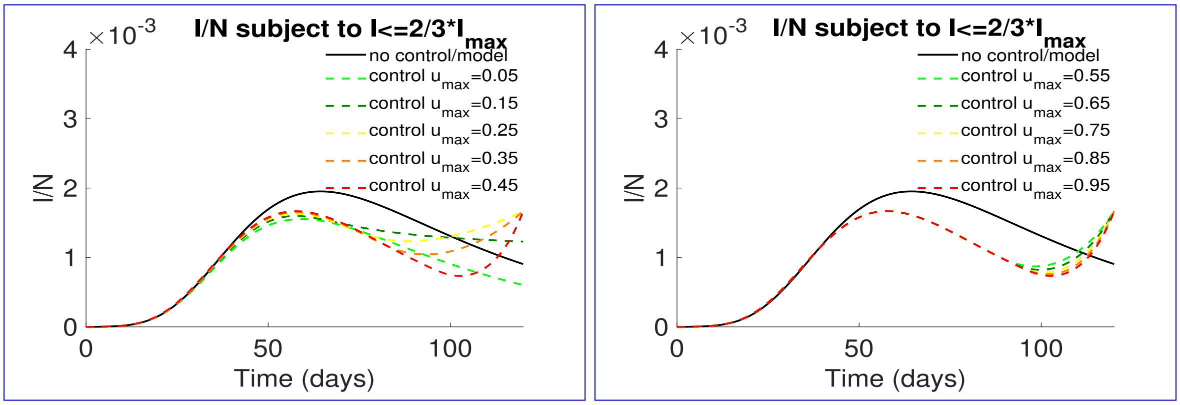


(a) (b)

**Supplementary Figure** 4: **Fraction of active infected individuals I/N.** With control (dotted colored lines) and without control (continuous black line). The controlled solutions are subject the constraint $I(t) \leqslant \frac{2}{3} \times I_{\max}$ and different values of $u_{\max} = \{0.05, 0.15, 0.25, 0.35, 0.45, 0.55, 0.65, 0.75, 0.85, 0.95\}$.

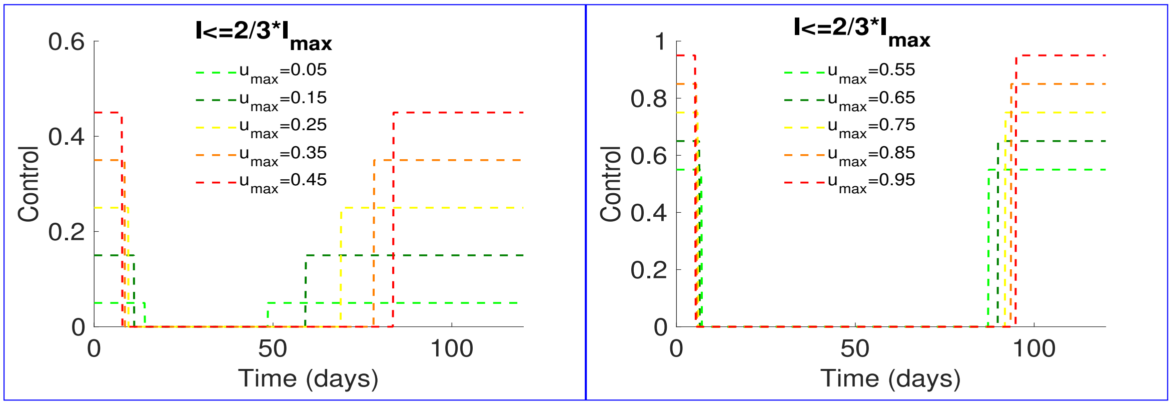


(a) (b)

**Supplementary Figure 5: Solution u of the optimal control problem.** Considering the state constraint $I(t) \leqslant \frac{2}{3} \times I_{\max}$ and different values of $u_{\max} = \{0.05, 0.15, 0.25, 0.35, 0.45, 0.55, 0.65, 0.75, 0.85, 0.95\}$.

Table 1: **Analysis of the time interval with no transfer from P to S ($\mathbf{u}_{\max} = \mathbf{0}$) after releasing $\mathbf{u}_{\max}$ individuals in the first period.** The control takes the maximum value $u_{\max}$, considering the constraint $I(t) \leqslant \frac{2}{3} \times I_{\max}$.

| Control $u(\cdot)$ | Time interval | Control $u(\cdot)$ | Time interval |
|---|---|---|---|
| $u_{\max} = 0.05$ | $\approxeq$ 34.4 days | $u_{\max} = 0.55$ | $\approxeq$ 79.8 days |
| $u_{\max} = 0.10$ | $\approxeq$ 42.6 days | $u_{\max} = 0.60$ | $\approxeq$ 81.8 days |
| $u_{\max} = 0.15$ | $\approxeq$ 47.9 days | $u_{\max} = 0.65$ | $\approxeq$ 83.3 days |
| $u_{\max} = 0.20$ | $\approxeq$ 52.1 days | $u_{\max} = 0.70$ | $\approxeq$ 84.6 days |
| $u_{\max} = 0.25$ | $\approxeq$ 59.4 days | $u_{\max} = 0.75$ | $\approxeq$ 85.8 days |
| $u_{\max} = 0.30$ | $\approxeq$ 65.3 days | $u_{\max} = 0.80$ | $\approxeq$ 86.8 days |
| $u_{\max} = 0.35$ | $\approxeq$ 69.6 days | $u_{\max} = 0.85$ | $\approxeq$ 87.8 days |
| $u_{\max} = 0.40$ | $\approxeq$ 73 days | $u_{\max} = 0.90$ | $\approxeq$ 88.6 days |
| $u_{\max} = 0.45$ | $\approxeq$ 75.7 days | $u_{\max} = 0.95$ | $\approxeq$ 89.5 days |
| $u_{\max} = 0.50$ | $\approxeq$ 78.1 days | | |

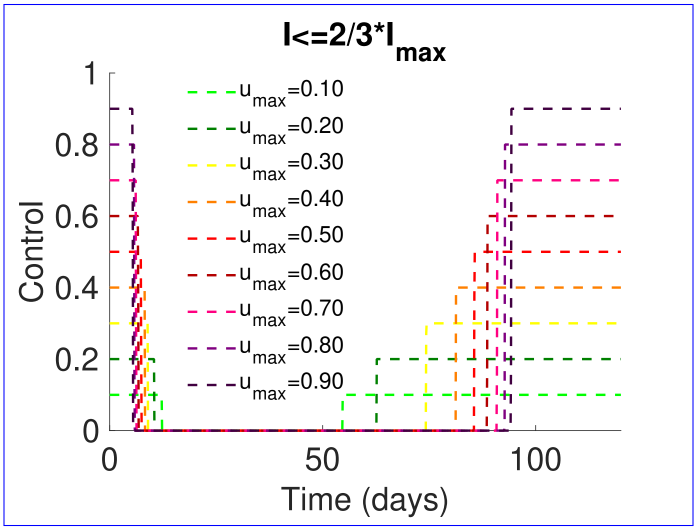


**Supplementary Figure** 6: **Solution of the optimal control problem u subject to the state constraint** $\mathbf{I(t) \leqslant \frac{2}{3} \times I_{\max}}$ **and different values of** $\mathbf{u_{\max} = \{0.10, 0.20, 0.30, 0.40, 0.50, 0.60, 0.70, 0.80, 0.90\}}$**.**

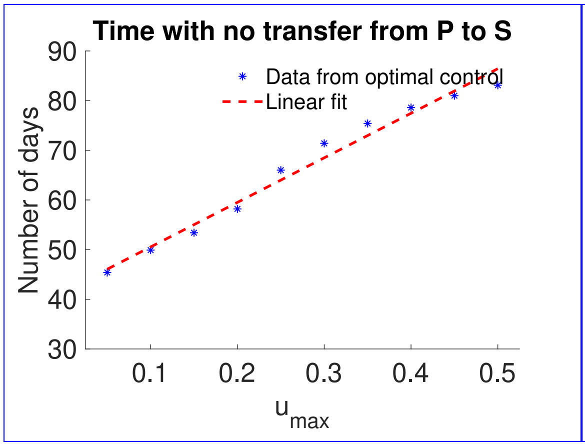


(a)

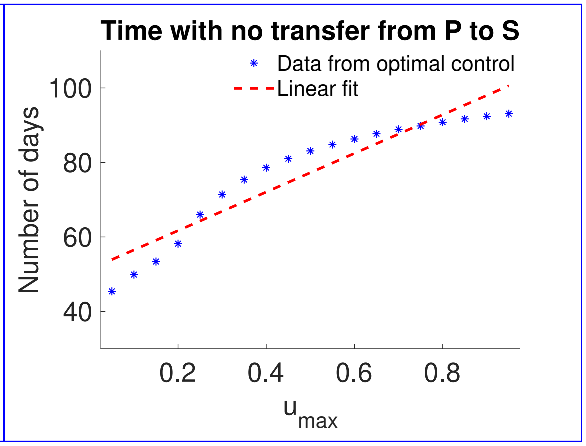


(b)

**Supplementary Figure** 7: **Time with no transfer from P to S subject to** $\mathbf{I \leqslant 0.60 \times I_{max}}$ **with a linear fit analysis.** Analysis of the relation/pattern between the maximal value $u_{\max}$ of the control and the number of days where there are no transfer of individuals from class $P$ to the class $S$, here referred as the *no transfer time interval*, after having released the fraction $u_{\max}$ of persons from class $P$ to class $S$. In (a) the discontinuous red line is obtained by the linear fit $y = 89.697x + 41.573$ for $u_1 \in [0; 0.50]$ and in (b) by $y = 51.863x + 51.326$ for $u_1 \in [0; 0.95]$, where $y$ corresponds to the number of days with no transfer of individuals from $P$ to $S$, after having released the fraction $x$ (equiv. $u$) of class $P$ to class $S$ (the linear fit is obtained by means of a standard linear regression procedure).

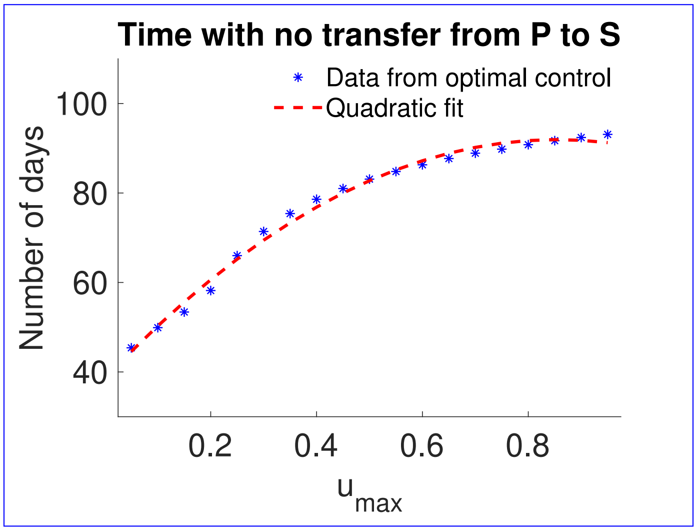


**Supplementary Figure** 8: **Time with no transfer from P to S subject to $\mathbf{I} \leqslant \mathbf{0.60} \times \mathbf{I}_{\max}$ with a quadratic fit analysis.** Analysis of the relation between the maximal value $u_{\max}$ of the control and the number of days that there are no transfer of individuals from class $P$ to the class $S$, considering a quadratic fit (red discontinuous line) $y = -73.251x^2 + 125.114x + 38.507$ and $u_{\max} \in [0; 0.95]$ w.r.t. time with no transfer from $P$ to $S$.

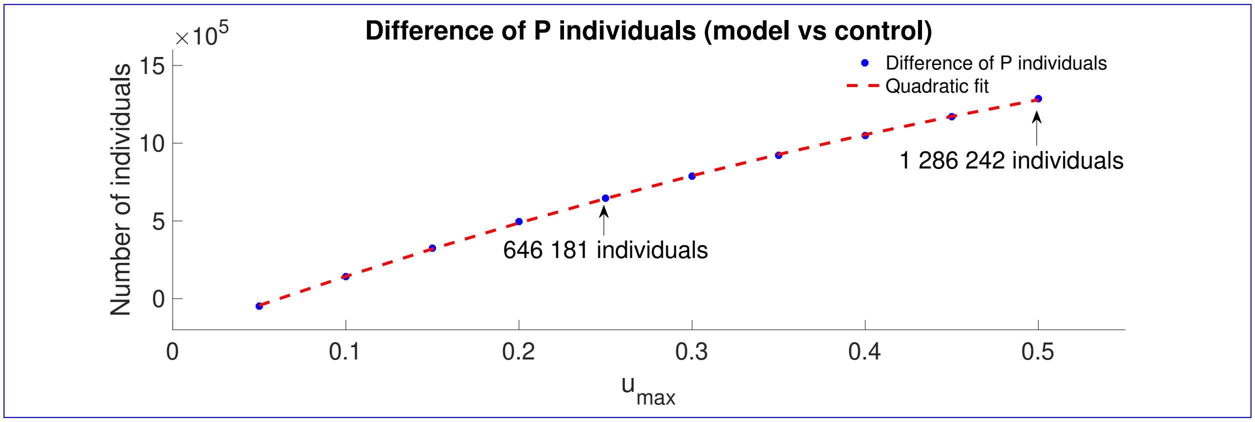


**Supplementary Figure** 9: **Difference between protected individuals obtained via the considered SAIRP model and the model with control**. Consider the maximal value of the control $u_{\max} \in \{0.05, 0.10, \ldots, 0.45, 0.50\}$ and the constraint $I(t) \leqslant 0.60 \times I_{\max}$. The quadratic equation for fitting the difference between the number of individuals in class $P$ obtained via de $SAIRP$ model without and with control $u_{\max} \in \{0.05, 0.10, \ldots, 0.45, 0.50\}$ (that is the number of released people from the protected class to the susceptible), respectively, is given by $y = -1984603.049\,x^2 + 4030952.677\,x - 239897.361$.

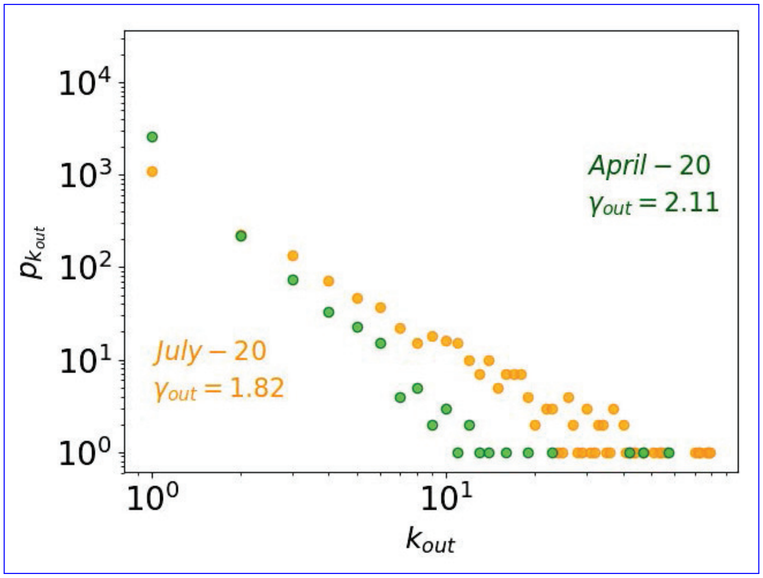


**Supplementary Figure** 10: **Connectivity distribution of the social networks obtained as described in the text.** Dots in green, as well as information in green, the network obtained in April 2020, while yellow dots correspond to the situation in July 2020. In both cases, the network topology corresponds to a scale free network with an exponent $\gamma = 2.11$ in April and $\gamma = 1.82$ in July 2020.
